# A Small Leak Detection Method Based on VMD Adaptive De-Noising and Ambiguity Correlation Classification Intended for Natural Gas Pipelines

**DOI:** 10.3390/s16122116

**Published:** 2016-12-13

**Authors:** Qiyang Xiao, Jian Li, Zhiliang Bai, Jiedi Sun, Nan Zhou, Zhoumo Zeng

**Affiliations:** 1 State Key Laboratory of Precision Measurement Technology and Instrument, Tianjin University, Tianjin 300072, China; yisuoyanyu058@tju.edu.cn (Q.X.); zhl_bai@tju.edu.cn (Z.B.); zhounan@tju.edu.cn (N.Z.); zhmzeng@tju.edu.cn (Z.Z.); 2 School of Information Science and Engineering, Yanshan University, Qinhuangdao 066004, China; sjdwjt@ysu.edu.cn

**Keywords:** pipeline small leakage detection, variational mode decomposition, adaptive de-noising method, ambiguity correlation classification

## Abstract

In this study, a small leak detection method based on variational mode decomposition (VMD) and ambiguity correlation classification (ACC) is proposed. The signals acquired from sensors were decomposed using the VMD, and numerous components were obtained. According to the probability density function (PDF), an adaptive de-noising algorithm based on VMD is proposed for noise component processing and de-noised components reconstruction. Furthermore, the ambiguity function image was employed for analysis of the reconstructed signals. Based on the correlation coefficient, ACC is proposed to detect the small leak of pipeline. The analysis of pipeline leakage signals, using 1 mm and 2 mm leaks, has shown that proposed detection method can detect a small leak accurately and effectively. Moreover, the experimental results have shown that the proposed method achieved better performances than support vector machine (SVM) and back propagation neural network (BP) methods.

## 1. Introduction

Due to increasing demands for natural gas, gas pipeline construction has developed rapidly. However, for a variety of natural and artificial reasons small leakage accidents happen occasionally in pipelines, which cause tremendous economic losses and casualties [1,2]. In order to provide for the safe operation of pipelines, small leak detection in pipelines should be studied [3,4]. Nowadays, pipeline leak detectors mainly determine the occurrence of leaking accidents based on delivery pressure and flow rate changes, which are influenced by various factors, such as transporting materials and transporting conditions [5,6]. When a leak occurs, there is an air-structure coupling between the high-velocity escaping gas and the tube wall surrounding the leak location, thereby causing stress waves that propagate along the body of the pipe on both sides of the leak hole. This phenomenon is known as stress wave emission and is considered to be a general acoustic emission. The acoustic emission (AE) technique realizes dynamic non-destruction testing according to AE source characteristics (e.g., defect type, size and position), which has advantages of high sensitivity and all-weather real-time detection [7,8,9]. Therefore, in this paper the AE technique was used for small leak detection in natural gas pipelines.

The complicated surrounding environment, big noise disturbances and non-stationary characteristics of collected signals in small pipeline leaks, require a signal de-noising method in order to extract the weak signal characteristics [10]. Traditional de-noising methods are based on noise frequency removal by a filter. However, this method has obvious limitations for signals with wideband noise, non-stationary signals and short-time transient signals [11,12]. The wavelet threshold de-noising method is superior to traditional de-noising methods, but it still cannot provide an ideal effect because of overlapping frequencies in the wavelet decomposition and the difficult selection of an accurate threshold [13,14]. Empirical mode decomposition (EMD) can reduce vibration signal noise, but EMD still has some theoretical problems, such as inadequate enveloping, over-enveloping, and mode mixing [15]. Vibrational mode decomposition (VMD) is a new non-stationary signal processing method proposed in 2014 [16]. VMD determines the frequency center and bandwidth of decomposed components by iteratively searching for the optimal solution for the variation mode, thus enabling an adaptive decomposition of the non-stationary signals [17]. Compared to EMD recursive “screening”, VMD decomposes signals into non-recursion and variation modes, and controls the convergences condition. Therefore, it can effectively eliminate the mode mixing in the decomposition process [18,19]. With respect to the non-stationary signals collected by sensors, this paper proposes the VMD adaptive de-noising method to process collected signals, extract noiseless component and eliminate noise disturbances.

Conventional pattern recognition methods, like the back propagation neural network (BP) and support vector machine (SVM), are widely applied in the non-destructive testing field. Riahi et al. distinguished signals of different corrosion stages by a BP neural network in the acoustic emission testing of a tank bottom [20]. Qu et al. classified three anomalous events of pipelines (leaking, digging and walking) using SVM and realized a pipeline leak detection method [21]. Nevertheless, the BP neural network has the disadvantages of abundant parameter settings, slow convergence and easily getting caught in a local minimum, which restricts its diagnosis accuracy and wide applicability [22]. Compared to the BP neural network, the SVM generalization performance has been improved greatly, but it requires artificial assignment of kernel functions and kernel function parameters, thus restricting SVM applications significantly. Ambiguity function is a traditional time-frequency analysis tool that has an important role in non-stationary signal analysis and processing theory. In addition, it has been widely used in radar signal analysis and processing, optical information processing, etc. [23]. However, the ambiguity function has problems with cross terms and difficulties extracting signal time-frequency characteristics. In order to solve these problems, the ambiguity correlation classifier (ACC) is constructed based on the correlation coefficient, which can reduce the computational load and avoid disturbances from cross terms, without parameter setting. ACC recognizes different signal types according to internal relationships of eigenvalues and provides a method for small leak detection in pipelines. In this paper, a pipeline leak detection method based on VMD and ACC is proposed for small leakage accidents in natural gas pipelines. Firstly, the collected signals were decomposed by VMD and the adaptive de-noising method was proposed for the de-noising process. Afterwards, noiseless components were reconstructed and de-noising signals were obtained. Finally, according to the signal ambiguity characteristics, ACC was proposed to train and test different types of reconstructed signals. Experimental results demonstrated that this method can detect small pipeline leak accidents accurately.

The paper is organized as follows: in Section 2, the proposed adaptive de-noising method based on VMD is described, and a simulation is presented in order to illustrate the proposed method. In Section 3, the ambiguity correlation classifier for pipe leakage detection is presented. In Section 4, the small leakage detection method is explained. The proposed leakage-detection scheme is experimentally validated, and compared with BP and SVM methods in Section 5. Lastly, brief conclusions are given in Section 6.

## 2. VMD Adaptive De-Noising Method

### 2.1. Variational Mode Decomposition

Variational mode decomposition is a new self-adaptive signal processing method, which was proposed by Dragomiretskiy in 2014 [16]. This method achieves self-adaptive signal decomposition through construction and solution of variational problems [17]. The VMD algorithm transfers the signal decomposition process to the variational framework. Hence, the VMD decomposition process is an optimal-solution processing for a constrained variational process. For constrained-variation problems, the augmented Lagrange function is introduced to transform the constrained variation problem into an unconstrained variation problem.

In order to obtain an optimal solution, the alternate direction method of multipliers (ADMM) is used to calculate the saddle point of the augmented Lagrange function, which is the optimal solution of the constrained-variation equation [18]. The saddle-point problem is solved by the alternate renewal of components and Lagrange multiplier, i.e., by solving the optimal-solution problem of the variational problem.

The VMD algorithm continuously updates the modes in the frequency domain, and finally transforms them to the time domain through the Fourier inversion transformation. The specific algorithm is as follows:

(1) Initialize $\left\{{\hat{u}}_{k}^{1}\right\}$, $\left\{\omega_{k}^{1}\right\}$, ${\overset{⌢}{\lambda }}^{1}$, and n=0. λ is the Lagrange multiplier, $\left\{u_{k}\}=\{u_{1},\ldots ,u_{k}\right\}$ represents K components set after decomposition, and $\left\{\omega_{k}\}=\{\omega_{1},\ldots ,\omega_{k}\right\}$ represents the center frequencies set of components after decomposition.
(2) n=n+1, execute the whole loop.
(3) Execute innermost loop, k=1:k, $u_{k}$ and $\omega_{k}$ are renewed according to ${\hat{u}}_{k}^{n+1}$ and $\omega_{k}^{n+1}$:
  (1) $$\left\{{\hat{u}}_{k}^{n+1}\right\}=\frac{\hat{f}(\omega )-\sum_{i<k}{\hat{u}}_{i}^{n+1}(\omega )-\sum_{i>k}{\hat{u}}_{i}^{n}(\omega )+({\hat{\lambda }}^{n}(\omega )/2)}{1+2\alpha {\left(\omega -\omega_{k}^{n}\right)}^{2}}$$
  (2) $$\omega_{k}^{n+1}=\frac{\int_{0}^{\infty }\omega {\left|{\hat{u}}_{k}^{n+1}(\omega )\right|}^{2}d\omega }{\int_{0}^{\infty }{\left|{\hat{u}}_{k}^{n+1}(\omega )\right|}^{2}d\omega }$$
  where α is the penalty parameter, f(t) represents the signal, and real part of $\left\{{\hat{u}}_{k}(\omega )\right\}$ conducting Fourier inversion is $\left\{u_{k}(t)\right\}$.
(4) Renew λ according to ${\overset{⌢}{\lambda }}^{n+1}(\omega )$ for ω≥0.
  (3) $${\hat{\lambda }}^{n+1}(\omega )={\hat{\lambda }}^{n}(\omega )+\tau [\hat{f}(\omega )-\sum_{k}{\hat{u}}_{k}^{n+1}(\omega )]$$
  where τ is the update parameter of the Lagrangian multiplier.
(5) If $\sum_{k}{\left\|{\hat{u}}_{k}^{n+1}-{\hat{u}}_{k}^{n}\right\|}_{2}^{2}/{\left\|{\hat{u}}_{k}^{n}\right\|}_{2}^{2}\leq \varepsilon$, end the whole loop, output the k modal components $u_{k}$, otherwise, repeat steps from Equation (2) to Equation (4).

### 2.2. VMD-Based Noise Adaptive Removal Approach

Signals collected by sensors often contain noise interference, which weakens the leakage characteristics. In order to extract the characteristic information accurately, components after VMD have to be de-noised, which increases the signal-to-noise ratio (SNR). Currently, most studies divide components after VMD into noise components and noiseless components. They eliminate noise components and reconstruct noiseless components to realize a de-noising process [24]. Sun et al. presented a multi-wavelet block threshold method to remove the noise components [25]. Kang et al. effectively eliminated high-level noise by exploiting a soft–threshold with adaptively estimated positive and negative noise levels from the time domain AE signal [14,26]. As it is well known, an amplitude is a signal characteristic, so noise information could be gained by amplitude analysis. Probability density function (PDF) means that the probability of some random vibration signal amplitude is within certain range [27]. Therefore, the PDF can be used to distinguish the noise signals. Hence, an adaptive de-noising method was proposed in this paper by combing VMD and PDF, which was used for signals processing and de-noising signals acquisition. Specific steps of the algorithm are:

(1) For any data $\left\{x_{i},i=1,\ldots ,n\right\}$, calculate its PDF ${\hat{f}}_{h}(x)$:
  (4) $${\hat{f}}_{h}(x)=\frac{1}{nh}\sum_{i=1}^{n}k(\frac{x-x_{i}}{h})$$
  where k(•) presents the Gaussian kernels function and h is the bandwidth. To prevent overlarge of variance and deviation h is often determined 0.15 times of the prediction confidence interval of variable *x*.
(2) Calculate PDFs of the original signal x(t) and component $u_{k}(t)$ after VMD:
  (5) P=PDF(X(t))
  (6) $$Q_{k}=PDF(u_{k}(t))$$
  where p and Q are PDFs, and K is the number of decomposed components.
(3) Calculate distance (L(k)) between two PDFs:
  (7) $$L(k)=dist(PDF(x(t)),PDF(u_{k}(t)))$$
  (8) $$\begin{array}{l} L(k)={\left\|P-Q\right\|}_{2}\\ =\sqrt{\int_{-\infty }^{+\infty }{\left(P(z)-Q(z)\right)}^{2}dz} \end{array}$$
(4) Choose noiseless component according to the distance:
  (9) $$N_{th}=\underset{1\leq k\leq K}{\arg\max\{L(k)\}}$$

It can be concluded from Equation (9) that $N_{th}-1$ components are noiseless components and the rest of them are noise components.

### 2.3. Algorithm Simulation

In order to verify the algorithm effectiveness, a simulation signal is used. The simulation signal was composed of frequency-modulated and amplitude-modulated signals and random white noises. The corresponding mathematical expressions are:

(10) $$x_{1}(t)=[1+0.3\cos(10\pi t)]\sin[200\pi t+\sin(15\pi t)]$$

(11) $$x_{2}(t)=\cos[60\pi t+\sin(10\pi t)]$$

(12) $$x(t)=x_{1}(t)+x_{2}(t)+0.4randn$$

(13) t=[0,0.4]

**Figure 1:**
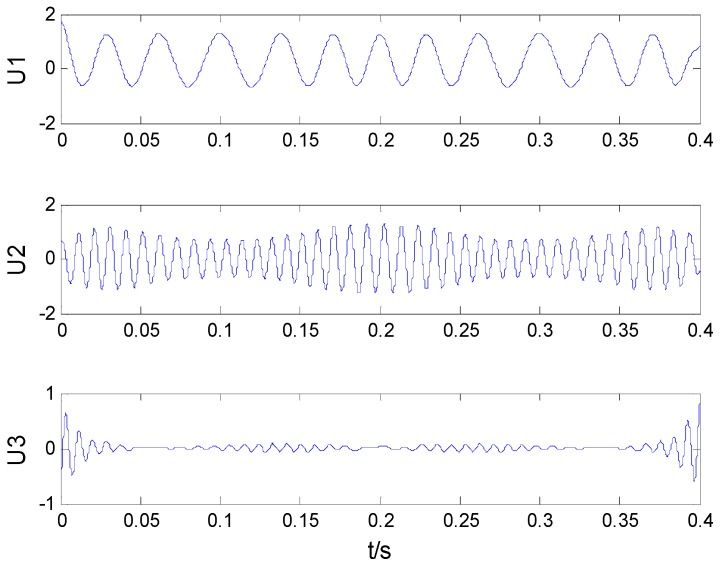
VMD results of the simulated signal.

**Figure 2:**
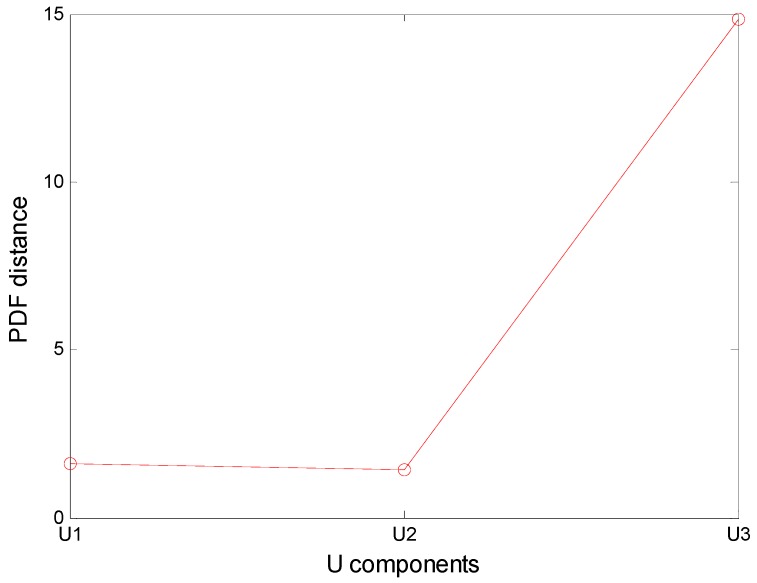
PDF distance between *U* components and the original signal.

After VMD of the simulation signal, PDFs of decomposed components and the original signal were calculated. VMD results are shown in Figure 1 and the distance between two PDF is shown in Figure 2.

In Figure 2, the PDF of *U*_3_ has the largest distance of the original signal. Therefore, previous two components were chosen as the noiseless components. According to Figure 1, two previous components are noiseless components and the third component is the noise component. The obtained results show that the VMD adaptive de-noising method can eliminate noise interference effectively and obtain noiseless signals.

Mean error square (MES) and SNR are often used to evaluate de-noising effect [28]. EMD and VMD were used for de-nosing process, and obtained results are listed in Table 1.

A higher SNR and a smaller MES after de-noised signal indicate a better de-noising effect. As it can be seen in Table 1, EMD has poor de-noising effects due to mode mixing, while VMD achieves good de-noising effects since it can decompose non-stationary signals effectively and acquire the correct components.

## 3. Ambiguity Correlation Classifier

### 3.1. Ambiguity Correlation Theory

As a non-stationary signal analysis method, the ambiguity function has been widely used due to its advantages in delay-frequency shift plane analysis [23]. Like Cohen’s time-frequency distribution, the ambiguity function suffers from a serious cross terms interference in signal analysis and has a heavy computational load. Interference disturbs effective analysis and signal explanation, as well as component parameters extractions, thus causing difficult extraction of the time-frequency signal characteristics. Hence, there must be an internal relationship between eigenvalues of different signal types. Such an internal relationship varies significantly among different types. Correlation coefficients can be used as the internal relationship measure. Different absolute values of correlation coefficients indicate different correlation degrees between signal eigenvalues [29]. According to that, the ambiguity correlation method was proposed in order to avoid interference from cross terms and to reduce the computation load of a time-frequency analysis. Specific steps are as follows:

(1) Calculate ambiguity function of signal *x*(*t*):
  (14) $$A(\tau ,\theta )=\frac{1}{2\pi }\int r_{x}(t,\tau )e^{j\theta t}dt$$
  (15) $$r_{x}(t,\tau )=x(t+\tau /2)x*(t-\tau /2)$$
  where $r_{x}(t,\tau )$ is the autocorrelation function of signals, A(τ,θ) is the ambiguity function of signal *x*(*t*).
(2) Calculate the correlation function of ambiguity function images of signals *x*(*t*) and *y*(*t*):
  (16) $$R_{xy}(\tau ,\theta )=\underset{\tau_{0},\theta_{0}}{\max}\left|\int_{-\infty }^{\infty }\int_{-\infty }^{\infty }A_{x}(\tau ,\theta )A_{y}(\tau -\tau_{0},\theta -\theta_{0})d\tau d\theta \right|$$
  In Equation (16), $R_{xy}(\tau ,\theta )$ is the correlation function.
(3) Calculate normalized correlation coefficient $\rho_{xy}(\tau ,\theta )$:
  (17) $$\rho_{xy}(\tau ,\theta )=\frac{\underset{\tau_{0},\theta_{0}}{\max}\left|\int_{-\infty }^{\infty }\int_{-\infty }^{\infty }A_{x}(\tau ,\theta )A_{y}(\tau -\tau_{0},\theta -\theta_{0})d\tau d\theta \right|}{{\left[\int_{-\infty }^{\infty }\int_{-\infty }^{\infty }A_{x}^{2}(\tau ,\theta )d\tau d\theta \int_{-\infty }^{\infty }\int_{-\infty }^{\infty }A_{y}^{2}(\tau ,\theta )d\tau d\theta \right]}^{\frac{1}{2}}}$$
(4) Select correlation coefficient when *τ* = 0 or *θ* = 0:
  (18) $$\rho_{xy}(0,\theta )=\frac{\underset{\tau_{0},\theta_{0}}{\max}\left|\int_{-\infty }^{\infty }\int_{-\infty }^{\infty }A_{x}(0,\theta )A_{y}(0-\tau_{0},\theta -\theta_{0})d\tau d\theta \right|}{{\left[\int_{-\infty }^{\infty }\int_{-\infty }^{\infty }A_{x}^{2}(0,\theta )d\tau d\theta \int_{-\infty }^{\infty }\int_{-\infty }^{\infty }A_{y}^{2}(0,\theta )d\tau d\theta \right]}^{\frac{1}{2}}}$$
  (19) $$\rho_{xy}(\tau ,0)=\frac{\underset{\tau_{0},\theta_{0}}{\max}\left|\int_{-\infty }^{\infty }\int_{-\infty }^{\infty }A_{x}(\tau ,0)A_{y}(\tau -\tau_{0},0-\theta_{0})d\tau d\theta \right|}{{\left[\int_{-\infty }^{\infty }\int_{-\infty }^{\infty }A_{x}^{2}(\tau ,0)d\tau d\theta \int_{-\infty }^{\infty }\int_{-\infty }^{\infty }A_{y}^{2}(\tau ,0)d\tau d\theta \right]}^{\frac{1}{2}}}$$
(5) Calculate the ambiguity correlation coefficient $\bar{\rho }$:
  (20) $$\bar{\rho }=\sqrt{\frac{\rho_{xy}^{2}(0,\theta )+\rho_{xy}^{2}(\tau ,0)}{2}}$$

Providing the reliable theoretical basis and method to build the identification system based on the ambiguity correlation coefficient of leakage signals.

### 3.2. Basic Principle of Classifier

**Figure 3:**
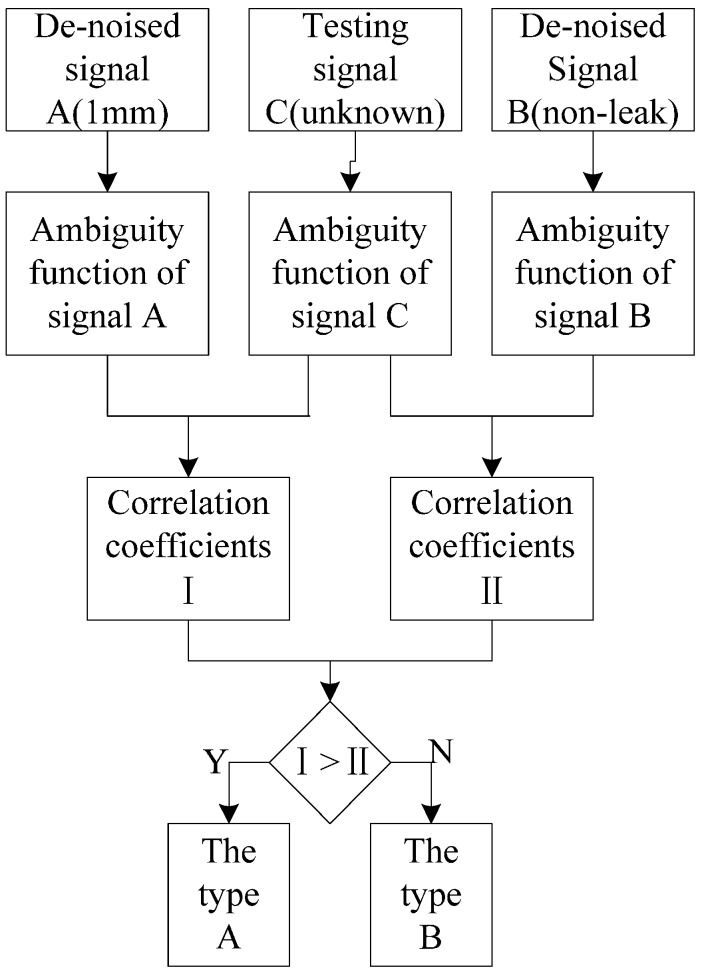
ACC flowchart.

The ambiguity correlation classifier (ACC) represents a one-to-one classifier. ACC has advantages of low computational load and no parameter setting. Firstly, two types of signals were processed by the VMD-based adaptive de-noising method in order to get the de-noised signals. Ambiguity functions of de-noised signals A and B, as well as the test signal C, were calculated. The correlation coefficient, I, between the ambiguity function of A and the ambiguity function of C was calculated. Similarly, the correlation coefficient, II, between the ambiguity function of B and the ambiguity function of C was calculated. Afterwards, I and II were compared. In the case that I > II, then C is equal to A; otherwise, C is equal to B. If A is a de-noised signal of a 1 mm leak, B is de-the noised signal of the non-leak situation, and C is an unknown signal. Next they are input into ACC to identify, and C can be determined to be a 1 mm leakage signal or no leakage signal. If C is neither a 1 mm leakage signal nor a non-leak signal, then C continues to be classified as described above. The principle of the ambiguity correlation classifier is shown in Figure 3.

## 4. Small Leak Detection Method

**Figure 4:**
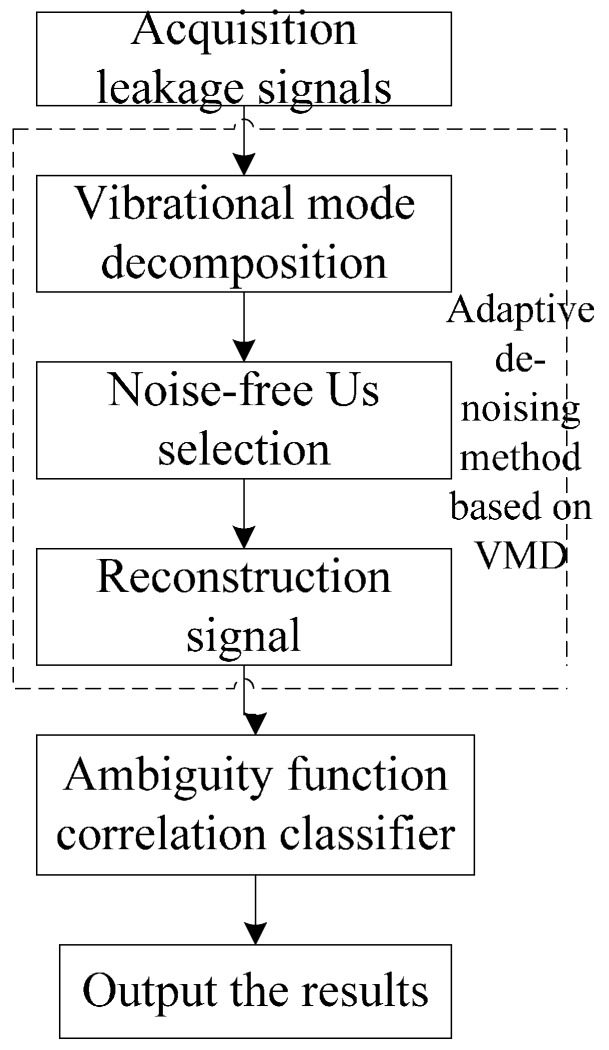
Flowchart of the small leakage detection method.

The small leak detection method based on VMD and ACC can eliminate noise interference, extract characteristic components from weak and input reconstructed signals into the classifier in order to realize a small leak detection. The flowchart of this detection method is shown in Figure 4. Specific steps are as follows:

(1) Collect experimental data by sensors and get series of *U* components of collected data from VMD.
(2) Select noiseless components according to VMD adaptive de-noising method.
(3) Reconstruct chosen noiseless components and input using the ACC.
(4) Collect several groups of data for training and testing, and realize the pipeline small leak detection.

## 5. Experimental Results and Analysis

### 5.1. Experimental Apparatus and Instrumentations

**Figure 5:**
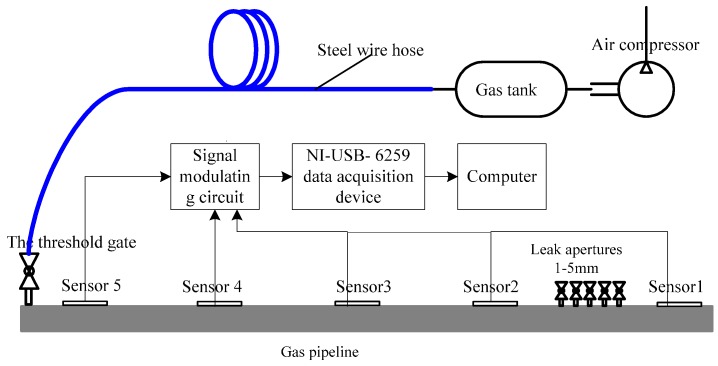
Experiment system schematic.

The experimental setup was composed of sensors, data acquisition cards, bearing pipelines and computer. The pipe was a seamless steel pipe with an outer diameter of 108 mm and wall thickness of 4.5 mm; and the leak testing was implemented based on 1 mm and 2 mm holes. Under the test pressure of 1.0–2.0 MPa, pipeline deflation was conducted at a flow rate of 3.1 m/s through internal decompression. The sensitivities of the YD-82 piezoelectric acceleration transducers used were 167.2 ${\mathrm{pC}/\mathrm{ms}}^{2}$ 165.8 ${\mathrm{pC}/\mathrm{ms}}^{2}$, 160.4 ${\mathrm{pC}/\mathrm{ms}}^{2}$, 189 ${\mathrm{pC}/\mathrm{ms}}^{2}$ and the frequency response is from 0 KHz to 10 KHz. The output was converted to a voltage signal by the charge amplifier. Experimental data were collected by a NI USB-6259 data acquisition card at a rate of 5 Ks/s. The experimental setup is shown in Figure 5.

**Figure 6:**
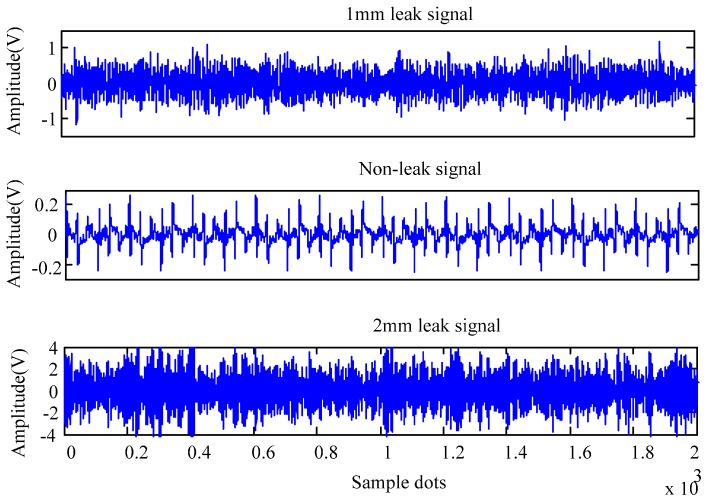
Signals collected by sensors.

Using the experimental platform, 1 mm leak signals, 2 mm leak signals and the signals under normal conditions (non-leak) were collected, and they are presented in Figure 6.

### 5.2. Acoustic Emission Signal De-Noising

**Figure 7:**
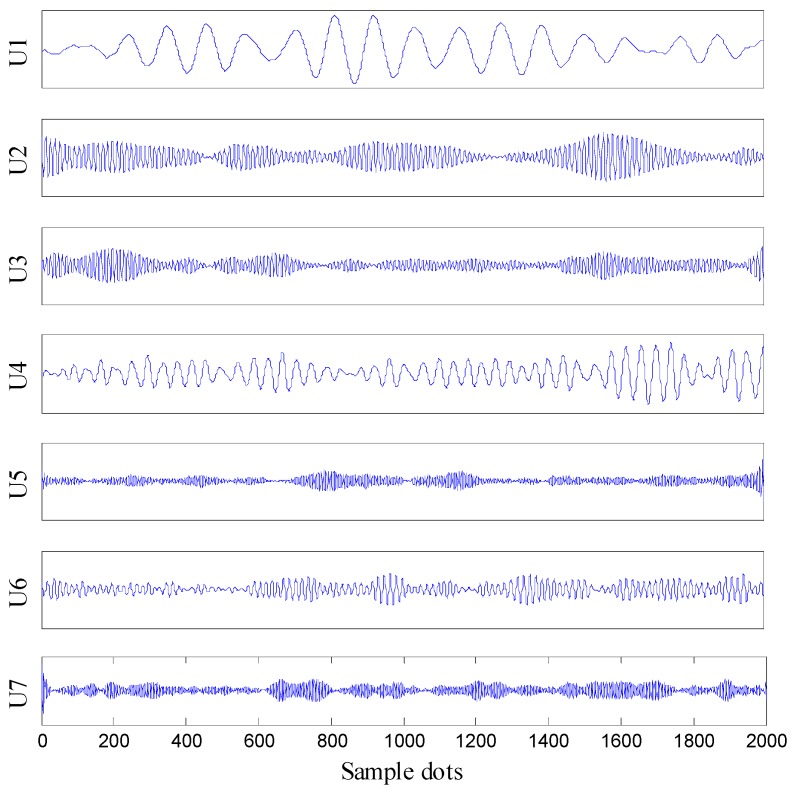
VMD results of a 1 mm leak signal.

The signal collected by one sensor under 1 mm leak was taken as an example. The obtained VMD results of that signal are shown in Figure 7.

**Figure 8:**
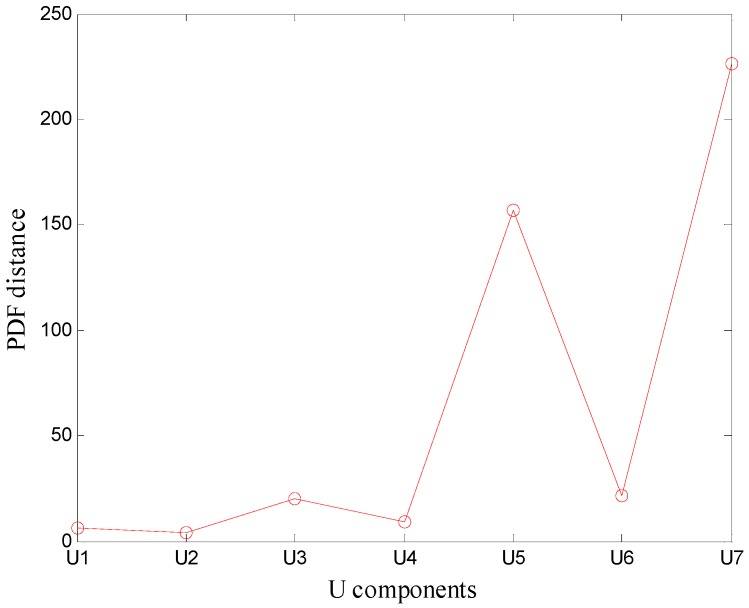
PDF distance between *U* components and the 1 mm leak signal.

Moreover, the signal components after VMD were processed by VMD adaptive de-noising method. The distances between the probability density function of the original signal and the probability density function of components were calculated (Figure 8).

**Figure 9:**
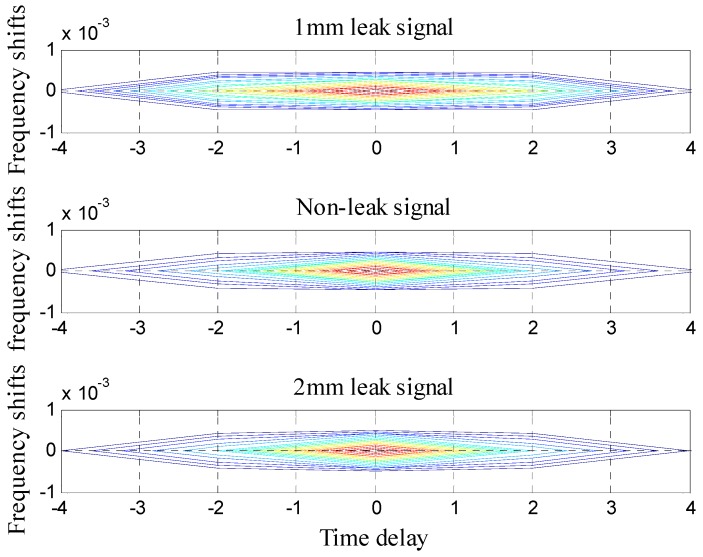
Ambiguity function images of de-noised signals.

As it can be seen in Figure 8, the probability density function of *U*_5_ has the largest distance of the original signal. Therefore, the first four components were chosen as the noiseless components and the remaining three were chosen as the noise components. The first four components were reconstructed, forming a reconstruction signal. Ambiguity function of the reconstructed signal was calculated and the result is presented in Figure 9.

In Figure 9, the ambiguity functions of three signals have similar delays and frequency shifts, so they could not be distinguished effectively. As a result, the ACC was proposed for extraction of these three signals’ characteristics and realization of small leak detection in pipelines.

### 5.3. Small Leakage Detection with ACC

For comparison purposes, mean values and standard deviations (SD) of the ambiguity correlation coefficients of de-noised signals based on VMD and EMD were calculated, and the obtained values are presented in Table 2 and Table 3, respectively.

**Figure 10:**
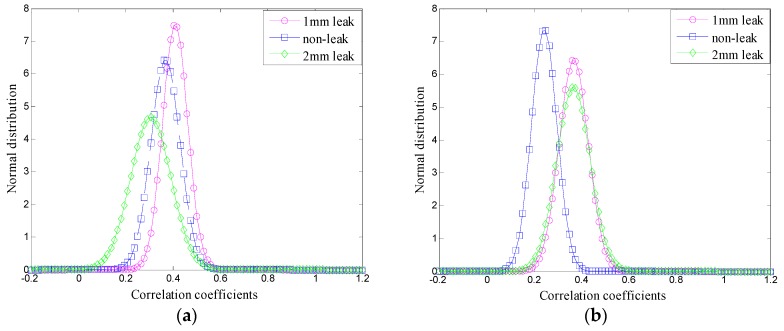

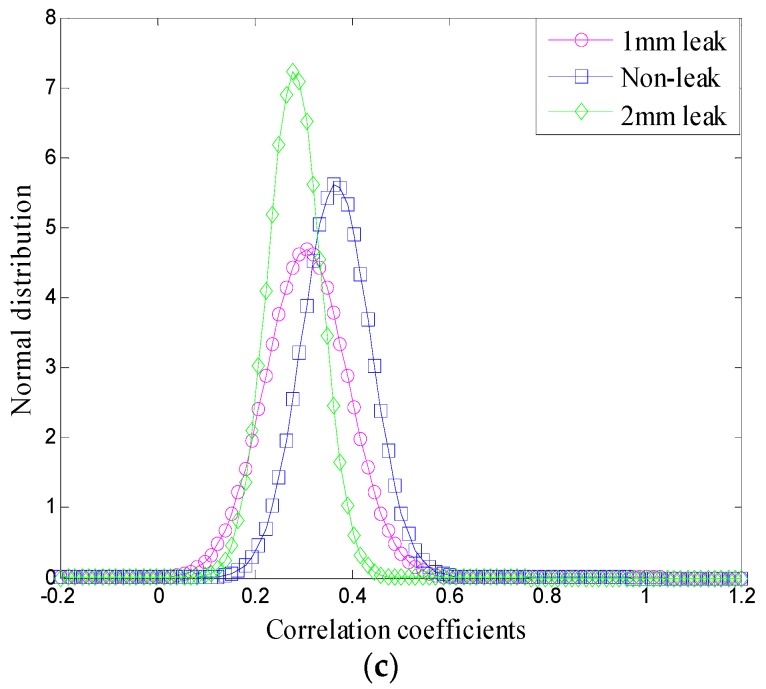
Normal distribution curves based on EMD: (**a**) for 1# case; (**b**) for 2# case; and (**c**) for 3# case.

**Figure 11:**
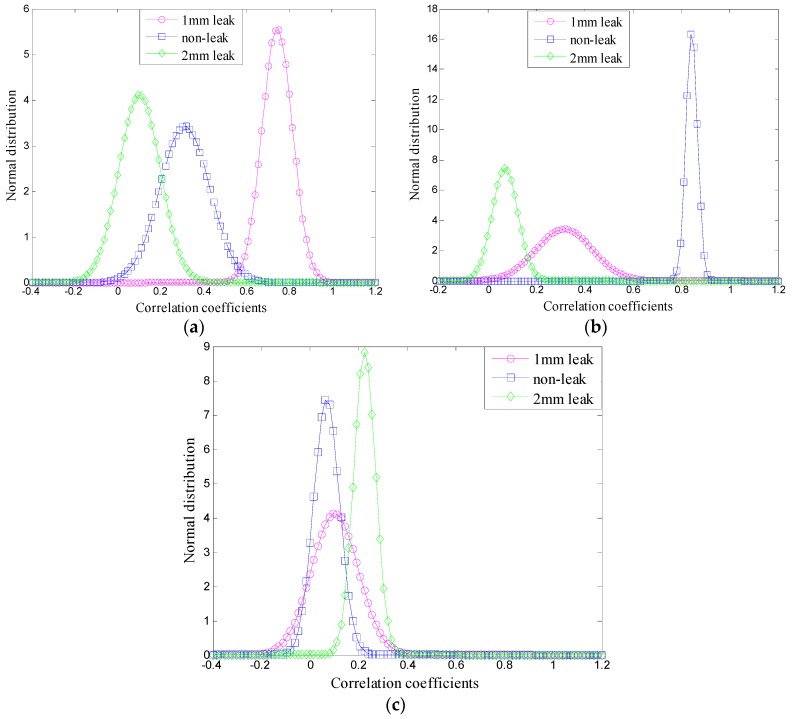
Normal distribution curves based on VMD: (**a**) for 1# case; (**b**) for 2# case; and (**c**) for 3# case.

According to results presented in Table 2, EMD could not acquire accurate components and eliminate noise interference due to the mode-mixing effect, resulting in a poor differentiation of mean values and SD of the ambiguity correlation coefficient. Thus, it is impossible to recognize different pipeline conditions. On the other hand, the VMD adaptive de-noising method can extract natural modes of vibration and separate noise components accurately. Therefore, the mean values and SD of the ambiguity correlation coefficients could be distinguished clearly. Further comparison was obtained by normal distribution curves of the correlation coefficients’ mean values and SD, presented in Table 2 and Table 3, and the obtained results are shown in Figure 10 and Figure 11, respectively.

Comparing the results presented in Figure 10 and Figure 11, it can be concluded that the normal distribution curves can intuitively present the correlation degree and recognition degree of the three signals in the ambiguity domain. Using the EMD-based de-noising method, the normal distribution curves could not be distinguished clearly because of the EMD mode mixing phenomenon, while the VMD can eliminate the mode mixing effectively in the decomposition process, thus, the VMD-based de-nosing method can distinguish the three states of the signals and the normal distribution curves.

**Figure 12:**
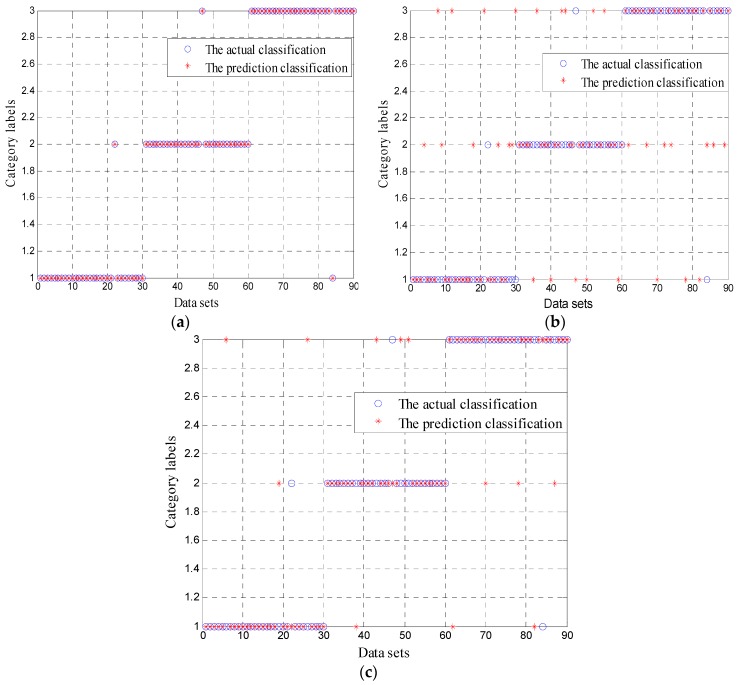
Classification of test figure: (**a**) obtained by ACC; (**b**) obtained by BP; and (**c**) obtained by SVM.

Thirty groups of data were chosen for training and testing, of which twenty groups were used for training, and ten groups were used for testing. Three classifiers, SVM, BP and ambiguity correlation classifier, were used for data training and testing. Test results are shown in Figure 12.

**Figure 13:**
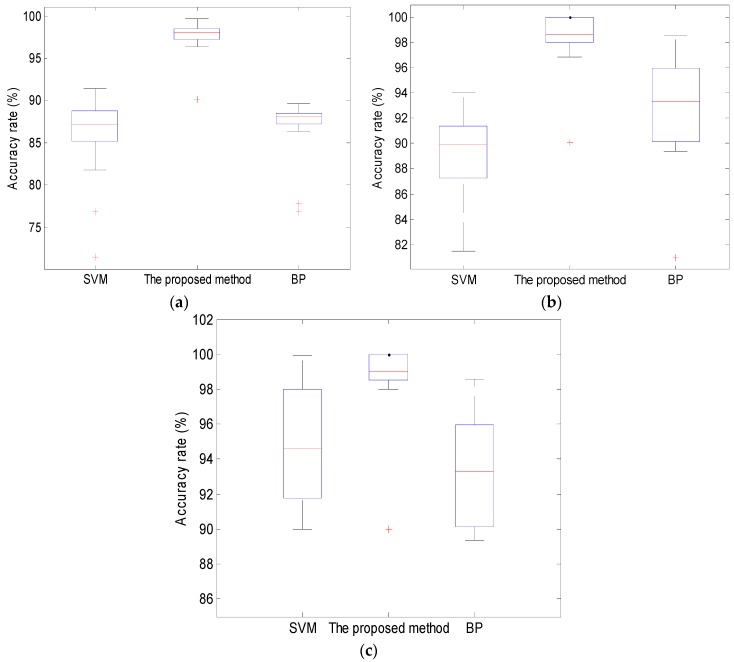
Test accuracy of classifiers: (**a**) for 1 mm leak; (**b**) for non-leak; and (**c**) for 2 mm leak.

In Figure 12, the training times of SVM, BP and ambiguity correlation classifier are 15 s, 25 s and 6 s, respectively. Compared to SVM and BP, the ambiguity correlation classifier achieves better classification effect and requires a shorter training time, which is caused by the absence of parameter setting and the small computational load. Test accuracies of these three classifiers for 60 groups of data, wherein 40 were used for training and 20 were used for testing, are presented in Figure 13.

As it can be seen in Figure 13, all three classifiers can distinguish the different conditions of pipelines, but compared to BP and SVM, the proposed classifier achieves the highest mean recognition rate and realizes the best small leak detection in pipelines, because of its small computational load and no parameter setting.

### 5.4. Discussion

The experimental results indicate that the proposed method can effectively detect small leaks in noisy environments. This improvement is due to the proposed VMD adaptive de-noising and ACC approach. Compared to the recursive “screening” mode of the EMD, VMD can non-recursively decompose a multi-component signals into a number of components and control the decomposition convergence conditions reasonably. Hence, the VMD adaptive de-noised method can effectively eliminate the mode-mixing phenomenon with good noise immunity. Due to its no parameter setting and small computational load, the ACC method achieves better classification effects and shorter training times than the BP or SVM methods. Despite the advantages offered by the proposed scheme for effectively detecting small leaks in natural gas pipelines, it has however certain limitations. The penalty parameters and number of components in the VMD decomposition process need to be prioritized, hence, our future research will focus on investigating how to adaptively set the parameters of the VMD method.

## 6. Conclusions

Due to the complicated surrounding environment, large noise disturbances, and weak signals during the small leakage of pipelines, pipeline-leakage signals have unobvious characteristics. A small leak detection method based on VMD and ACC is proposed in this paper. For the non-stationary signals with strong noise, the VMD adaptive de-noising method is proposed according to the signal probability density function in order to process the signal and to acquire the noiseless components. Noiseless components were then reconstructed successfully, forming the de-noised signals. Furthermore, in order to overcome the problems of traditional classifiers, such as heavy computational load and abundant parameter settings, the ambiguity correlation classifier is proposed for training and testing of the reconstructed signals. According to obtained experimental results, the proposed method can detect small pipeline leakages and achieve a higher mean detection rate than the classical SVM and BP methods.

## Figures and Tables

**Table 1:** MSE and SNR of simulated signals.

Method	Simulated Signals	MSE	SNR
	Before de-noising	0.0463	13.4451
VMD	After de-noising	0.004	24.0253
EMD	After de-noising	0.0234	17.5643

**Table 2:** Mean values and SD of ambiguity correlation coefficients using EMD method.

Data Group		1#	2#	3#
		1 mm Leak	Non-Leak	2 mm Leak
1 mm leak	mean value	0.4074	0.3664	0.3042
	SD	0.0530	0.0629	0.0969
Non-leak	mean value	0.3664	0.2415	0.3650
	SD	0.0629	0.0340	0.0534
2 mm leak	mean value	0.3042	0.3650	0.2789
	SD	0.0969	0.0534	0.0452

**Table 3:** Mean values and SD of ambiguity correlation coefficients using VMD method.

Data Group		1#	2#	3#
		1 mm Leak	Non-Leak	2 mm Leak
1 mm leak	mean value	0.7447	0.3134	0.1025
	SD	0.0717	0.1164	0.0969
Non-leak	mean value	0.3134	0.8407	0.0685
	SD	0.1164	0.0240	0.0534
2 mm leak	mean value	0.1025	0.0685	0.2253
	SD	0.0969	0.0534	0.0452
